# How does prolonged tennis playing affect lower limb muscles' activity during first and second tennis serves?

**DOI:** 10.1002/ejsc.12199

**Published:** 2024-09-21

**Authors:** Clint Hansen, Caroline Teulier, Jean‐Paul Micallef, Grégoire P. Millet, Olivier Girard

**Affiliations:** ^1^ Department of Neurology University Hospital Schleswig‐Holstein Kiel Germany; ^2^ CIAMS Université Paris‐Saclay Orsay France; ^3^ CIAMS Université d'Orléans Orléans France; ^4^ Faculty of Sport Science University of Montpellier Montpellier France; ^5^ Institute of Sport Sciences University of Lausanne Lausanne Switzerland; ^6^ School of Human Science (Exercise and Sport Sciences) The University of Western Australia Perth Western Australia Australia

**Keywords:** fatigue, lower limbs, racket sports, surface electromyography, tennis serve

## Abstract

We examined the effect of prolonged tennis playing on lower limb muscles' activity during the execution of first and second tennis serves. Ten male competitive tennis players executed five first and second serves before (pretest) and after (posttest) a 3‐h tennis match. Surface electromyographic (EMG) activity of four lower limb muscles (*vastus lateralis*, *rectus femoris*, *gastrocnemius lateralis*, and *soleus* muscles) on each leg was recorded along with maximum ball velocity measured by a radar gun and peak vertical forces recorded by a force platform. For the *vastus lateralis*, *gastrocnemius lateralis*, and *soleus* muscles of the left leg as well as the *vastus lateralis* muscle of the right leg, EMG amplitude decreased from pre‐ to posttests (*p* ≤ 0.033). These reductions in the EMG signal were generally more pronounced in the first serve (i.e., ranging from −10% to −40%) compared to the second serve (0% to −25%). Maximum ball velocity for both first (159 ± 12 vs. 154 ± 12 km/h) and second (126 ± 20 vs. 125 ± 15 km/h) serves remained unchanged from pre‐ to posttests (*p* = 0.638) Similarly, peak vertical forces did not differ between pretest and posttest for both first (1.78 ± 0.30 vs. 1.72 ± 0.29 body weight) and second (1.62 ± 0.25 vs. 1.75 ± 0.23 body weight) serves (*p* = 0.730). In conclusion, a 3‐h tennis match led to decreased activation levels in various leg muscles during serves, particularly in first serves compared to second serves. Despite consistent maximum ball velocity and peak vertical forces, these reductions in EMG signals suggest that skilled tennis players may adopt compensatory strategies after prolonged play.

## Highlights

- A 3‐h tennis match resulted in reduced relative electromyographic (EMG) amplitudes for various knee extensor and plantar flexor muscles on both sides during the serve.
- Reductions in EMG signals were more prominent in the first serve (−10% to −40%) compared to the second serve (0% to −25%).
- Despite unchanged maximum ball velocities and peak vertical forces, muscle recruitment adjustments occurred in the *vastus lateralis*, *gastrocnemius lateralis*, and *soleus* muscles in the left leg and the *vastus lateralis* muscle in the right leg.
- Understanding changes in lower extremity muscle activation following prolonged play can help players and coaches develop strategies, such as targeted strength and conditioning protocols, to maintain effective serve technique.

## 1 INTRODUCTION

Fatigue is a complex phenomenon marked by diminished force generation and/or an inability to sustain the required exercise intensity (Enoka & Duchateau, 2016). In tennis, lower extremity muscles play a crucial role in dynamic movements, encompassing repeated accelerations, decelerations, turns, and jumps during intermittent exercise bouts within a match (Reid & Duffield, 2014). Previous research has demonstrated that maximal isometric voluntary force in both knee extensors (Girard et al., 2008) and plantar flexors (Girard et al., 2011), along with accompanying surface electromyographic (EMG) activity, decreases after 3‐h tennis matches. Despite strength losses and reductions in leg stiffness observed after prolonged play in isolated leg muscle contractions and multi‐rebound jumps, they may not necessarily reflect alterations in lower limb engagement during functional movements such as tennis strokes (Wilson & Murphy, 1996). There is a widespread belief that the onset of fatigue negatively impacts racket skill performance, leading to missed strokes, poor timing, or difficulty in maintaining proper positioning (Reid & Duffield, 2014).

Several studies have explored how stroke quality is altered during simulated tennis exertion (Davey et al., 2002; Vergauwen et al., 1998). For instance, Davey et al. (2002) found that during a 35‐min exhaustive tennis simulation test, hitting accuracy of ground strokes gradually decreased, reaching a 69% reduction from the initial accuracy. Similarly, serve accuracy to the right court declined by ∼30%. However, these investigations were confined to protocols (i.e., returning balls projected by a machine to target zones) that may not accurately mimic actual tennis demands. To date, only few studies have examined the neuromechanical responses to prolonged playing during the tennis serve in realistic scenarios such as extended match play (Hornery et al., 2007). After a 3‐h tennis match, alterations in serve maximal angular velocities, joint kinetics, and decreased EMG activity levels during isolated isometric muscle actions of upper limb muscles were observed (Martin et al., 2016). However, the relevance of EMG measurements during localized muscle contractions, where muscle actions and joint positions differ considerably from an actual tennis serve, is questionable.

Because the sequential motion of body segments is influenced and ultimately determined by muscular forces, which decrease after extended play (Girard et al., 2008, 2011), distinctive patterns of lower limb involvement may emerge during serving (Colomar, Corbi, & Baiget, 2022; Colomar, Corbi, Brich, & Baiget, 2022). Moreover, after prolonged tennis playing, lower limb drive assessed from peak vertical force varied depending on the serve type, whereas maximum ball velocity remained unchanged (Girard et al., 2012). This indicates a potential adjustment in intersegmental coordination to maintain serve efficiency after prolonged tennis playing. In a study by Fenter et al. (2017), three‐set tennis match induced reductions in knee flexion angle and EMG amplitudes in the *biceps femoris* and *rectus femoris* muscles during the tennis serve (Fenter et al., 2017). However, it is noteworthy that data were collected for the back leg only and the type of serve was not controlled for. To date, it remains unclear whether prolonged tennis playing alters lower limb drive measured from surface EMG during the serve and the influence of the type of serve.

This study aimed to examine the effect of a 3‐h tennis match on the muscle activity of lower limbs during the execution of first and second tennis serves. We hypothesized that several leg muscles on both sides would exhibit reduced activation levels following prolonged play and that first serves would yield greater EMG values compared to second serves.

## 2 METHODS

### 2.1 Participants

Ten right‐handed male competitive players (mean ± SD: age 26.1 ± 4.7 years; height 181.5 ± 6.8 cm; and body mass 76.3 ± 7.6 kg), competing at regional to national levels, participated in the study. Participants were classified as “Tier 2: Trained/Developmental” according to the participant classification framework proposed by McKay et al. (2022). They were eligible if they had no history of injury to the lower extremities or any other body parts within the past year. The experiment was approved by the local ethics committee and conformed to the current Declaration of Helsinki guidelines.

### 2.2 Experimental design

Participants commenced the session with a general warm‐up, involving ∼5 min of jogging, followed by ∼5 min of athletic drills, including heel flicks, high knee runs, coordination skips, hopping, and progressive accelerations. Surface EMG electrodes were then applied to the skin (see “Electromyographic recordings”). A standardized 15‐min tennis warm‐up ensued, involving baseline play and typical tournament‐like practice serves. Subsequently, participants were instructed to perform various serves in a randomized order as part of the pretest battery (see “Tennis serves”). Each trial included the recording of EMG signals from four muscles on each leg (eight muscles in total), along with maximum ball velocity and peak vertical force. After completing a test battery (pretests), participants engaged in a 3‐h competitive tennis match against a similarly skilled opponent on an outdoor (Greenset^®^) tennis court. Scoring and time adhered to International Tennis Federation rules, allowing a maximum of 20 s rest between rallies, 90 s between changeovers, and 120 s between sets. Participants were directed to play at their optimal level, resembling an official tournament. Heart rate was continuously recorded and averaged every 5 s during the match using short‐range radio telemetry (S610; Polar Electro Oy, Kempelem). Immediately following the match (posttest), participants repeated the tennis serve test battery.

### 2.3 Tennis serves

All serves (first and second) were executed from the “deuce” or right service court within an indoor tennis facility. Specifically, participants were directed to target a 1 × 1 m area adjacent to the “T” of the right service box, maintaining a match pace. Five “successful” trials were collected as judged by a professional coach. For first serves, participants were required to minimize spin on the ball whereas, second serves were expected to feature a combination of topspin and sidespin (Girard et al., 2010). All participants were right‐handed and used their own rackets (with slight variations in mass, string, tension, and flexibility) to ensure comfort in executing each serve. Throughout all trials, participants adopted a ready position with both feet (front: left leg and back: right leg) located on a force platform (surface area: 100 × 80 × 7 cm) until the take‐off instant. Participants were instructed to strike the ball as they would in an official competition. The force platform, installed on the right side of the baseline, monitored peak vertical forces at a sampling frequency of 500 Hz (MP 100A‐CE, Biopac). Maximum ball velocity was recorded using a radar gun (Stalker ATS) fixed on a 2.5‐m high tripod, 2 m behind the players.

### 2.4 Electromyographic recordings

Muscle activity was recorded using surface electrodes (Bagnoli 8‐EMG system, Delsys) attached to eight muscles, including the *vastus lateralis*, *rectus femoris*, *gastrocnemius lateralis* and *soleus*, for each leg. The placement of electrodes followed SENIAM's recommendations (Hermens et al., 2000). Before fixing the electrodes, the skin was shaved, lightly abraded, and cleaned with alcohol to reduce impedance. Electrodes and cables were securely fastened to the skin with an elastic cohesive bandage to reduce movement artifacts. Participants kept the electrodes on their skin throughout the entire experiment; however, the position of the electrodes was marked because some participants needed replacements due to excessive sweating. The EMG signals were pre‐amplified near the electrodes (×1000), subjected to band‐pass filtering (high‐pass 20 and low‐pass 450 Hz), and full‐wave rectified. To obtain the EMG envelope, the raw data were processed by applying full‐wave rectification and a fourth‐order Butterworth low‐pass filter with a cut‐off frequency of 5 Hz (Hansen et al., 2017).

The peak EMG amplitudes recorded during first and second serves from the pretest battery were retained and considered as the reference, that is, representing the maximal level of activity, regardless of when the moment of the EMG peak occurred (Le Mansec et al., 2018). Subsequently, the EMG amplitude was averaged for each muscle and each serve type over the five successful trials. Finally, the magnitude of the EMG signals was compared after normalization using the peak EMG values (Hansen et al., 2023).

### 2.5 Statistical analysis

Data analysis was conducted using JASP Team 2023 (Version 0.17.2). The Shapiro–Wilk test was performed due to the sample size, indicating a significant departure from normality for the distribution of variables (*p* < 0.01). Consequently, nonparametric Kruskal–Wallis tests were employed for the EMG variables, peak vertical force, and maximum ball velocity. Dunn's Tests with Bonferroni‐adjusted *p*‐values were conducted when a significant main effect was identified. Cohen's effect sizes *d*_*z*_ are reported for follow‐up tests, with *small*, *moderate*, and *large* effects considered for *d*_*z*_ ≥ 0.2, *d*_*z*_ ≥ 0.5, and *d*_*z*_ ≥ 0.8, respectively (Cohen, 1988). Statistical significance was set at *p* < 0.05.

## 3 RESULTS

**FIGURE 1:**
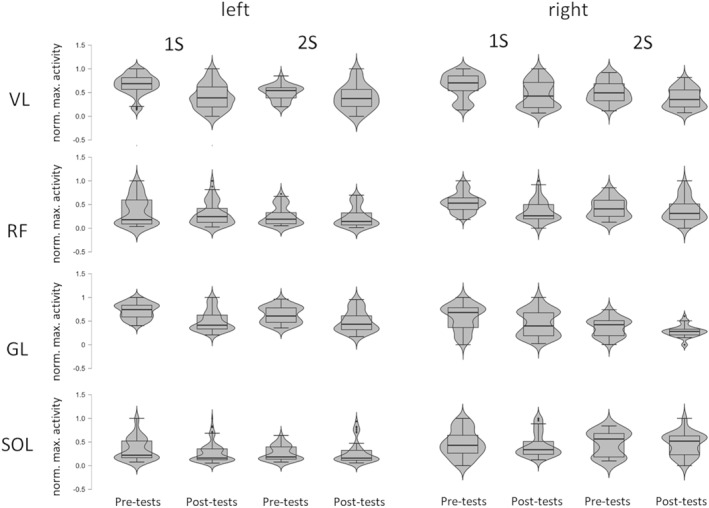
Violin plots visualizing surface electromyographic activity of eight muscles during the first (1S) and the second (2S) tennis serves before (pretests) and after (posttests) a 3‐h tennis match. Surface electromyography activity (EMG) for four muscles (*vastus lateralis* [VL], *rectus femoris* [RF], *gastrocnemius lateralis* [GL], and *soleus* [SOL]) of the left (left panels) leg and the right (right panels) leg was normalized to peak values obtained during actual tennis serves.

For the *vastus lateralis*, *gastrocnemius lateralis*, and *soleus* muscles of the left leg and the *vastus lateralis* muscle of the right leg, EMG amplitude decreased from pre‐ to posttests (*p* ≤ 0.033) (Figure 1 and Table 1). The left *rectus femoris* and *gastrocnemius lateralis* muscles and the right *vastus lateralis* and *gastrocnemius lateralis* muscles exhibited a main effect of serve type (*p* ≤ 0.037), with higher EMG levels for first than second serves.

**TABLE 1:** Results of statistical analysis.

Variables	First serve		Second serve		Kruskal–Wallis *p* value (*η* ^2^) [Cohen's *d*]	
(au)	Pretests	Posttests	Pretests	Posttests	Fatigue	Service type
Left leg						
EMG_VL_	0.657 ± 0.247	0.468 ± 0.282	0.512 ± 0.222	0.378 ± 0.208	0.033 (0.10) [−0.68]	0.092 (0.21) [0.30]
EMG_RF_	0.546 ± 0.195	0.349 ± 0.218	0.418 ± 0.206	0.371 ± 0.245	0.757 (0.032) [0.38]	0.020 (0.09) [0.64]
EMG_GL_	0.582 ± 0.271	0.458 ± 0.272	0.472 ± 0.264	0.450 ± 0.239	0.005 (0.01) [−0.82]	0.015 (0.01) [0.87]
EMG_SOL_	0.463 ± 0.278	0.423 ± 0.251	0.377 ± 0.191	0.283 ± 0.123	0.006 (0.01) [−0.03]	0.122 (0.04) [0.32]
Right leg						
EMG_VL_	0.672 ± 0.212	0.416 ± 0.265	0.498 ± 0.161	0.402 ± 0.263	0.028 (0.06) [−0.53]	0.037 (0.09) [0.65]
EMG_RF_	0.369 ± 0.304	0.298 ± 0.242	0.260 ± 0.199	0.233 ± 0.209	0.216 (0.01) [−0.22]	0.286 (0.01) [0.23]
EMG_GL_	0.718 ± 0.163	0.499 ± 0.217	0.628 ± 0.175	0.491 ± 0.222	0.694 (0.03) [0.33]	0.017 (0.06) [0.49]
EMG_SOL_	0.346 ± 0.241	0.263 ± 0.199	0.258 ± 0.155	0.260 ± 0.222	0.329 (0.12) [0.74]	0.160 (0.09) [0.66]

*Note*: Surface electromyography activity (EMG) for four muscles (*vastus lateralis* [VL], *rectus femoris* [RF], *gastrocnemius lateralis* [GL], and *soleus* [SOL]) of the left leg and the right leg was normalized to peak values obtained during actual tennis serves. Kruskal–Wallis test for fatigue and service type are stated along with partial eta squared (*η* ^2^) and [Cohen's *d*].

Maximum ball velocity for both first (159 ± 12 vs. 154 ± 12 km/h) and second (126 ± 20 vs. 125 ± 15 km/h) serves remained unchanged from pre‐ to posttests (*p* = 0.638 and *d* = 0.16). Maximum ball velocity was faster for first than second serves (*p* < 0.001 and *d* = 2.06). Peak vertical forces did not differ between pretest and posttest for both first (1.78 ± 0.30 vs. 1.72 ± 0.29 body weight) and second (1.62 ± 0.25 vs. 1.75 ± 0.23 body weight) serves (*p* = 0.730 and *d* = −0.12). Peak vertical forces were not different between first and second serves (*p* = 0.502 and *d* = 2.23).

During the 3‐h tennis match, the mean heart rate was 145 ± 10 beats/min (range: 129–166), indicating a mean intensity of 73 ± 5% of the maximal heart rate (range: 65–84) estimated as 220–age. Peak heart rate values reached 180 ± 13 beats/min (range: 162–200).

## 4 DISCUSSION

### 4.1 Summary of main findings

Our main observation reveals that prolonged tennis playing led to lower relative EMG amplitudes for several leg muscles during the serve, despite unchanged maximum ball velocities and peak vertical forces. Notably, these reductions in the EMG signal were more prominent in the first serve (i.e., ranging from −10% to −40%) compared to the second serve (i.e., ranging from 0% to −25%). These findings support our initial hypothesis, suggesting that several leg muscles on both sides experience reduced activation levels following prolonged play, with first serves demonstrating larger substantial EMG reductions than second serves.

### 4.2 Effects of prolonged tennis playing

There was a reduction in activity levels for several muscles for a 3‐h tennis match, regardless of the serve type. Specifically, these alterations in the EMG signal were statistically significant for the *vastus lateralis*, *gastrocnemius lateralis*, and *soleus* muscles in the left leg as well as for the *vastus lateralis* muscle in the right leg. This suggests that both knee extensors and plantar flexors were less actively engaged while serving due to prolonged tennis play. As far as we are aware, a limited number of prior surface EMG studies have investigated alterations in lower limb activity on both legs after extended tennis play, particularly when comparing first and second serves. In a scoping review by Brito et al. (2023), out of 91 studies, only 13 reported results on surface EMG, with the majority focusing on muscles in the hitting arm and shoulder region. Surface EMG activity of lower limb muscles was mainly examined under “fresh” conditions (Girard et al., 2005; Mourtzios et al., 2021; Rota et al., 2014), so that prolonged tennis playing effects remain unclear. In one study, a decrease in EMG amplitude was observed in the *rectus femoris* and *biceps femoris* muscles during a three‐set tennis match (Fenter et al., 2017). Interestingly, reductions in EMG signals were already visible at the end of the first set, with limited further changes thereafter. Throughout a 3‐h tennis match, progressive reductions in maximal voluntary strength were observed in the quadriceps (Girard et al., 2008), whereas strength loss in plantar flexors followed a more biphasic pattern and became more pronounced in the second half of the game (Girard et al., 2011). In the present study, as serves were only assessed before and after (i.e., within minutes) match play, it cannot be ruled out that the time course of changes in EMG activity throughout the match may have differed across muscle groups.

When interpreting EMG findings, it is important to consider that there was no significant reduction in maximum ball velocity for both first and second serves. Previously, mixed results have been reported regarding the effects of extended play on maximum ball velocity during the serve, with some studies showing unchanged (Colomar, Corbi, & Baiget, 2022; Colomar, Corbi, Brich, & Baiget, 2022; Maquirriain et al., 2016; Moreno‐Pérez et al., 2019) or decreased (Martin et al., 2016; Mitchell et al., 1992; Rota et al., 2014) values. A key finding of the present study consists in the lack of decrease in the magnitude of peak vertical forces and even a small nonsignificant increase for the second serve. These observations align with findings from Martin et al. (2016) who showed that two lower body serve variables (i.e., maximal back knee flexion angle and maximal back knee extension angular velocity) were significantly different across time whereas, peak vertical force remained unchanged. Importantly, the intensity of match play was comparable to that of match simulations involving players of similar standard (Girard et al., 2008, 2011), indicating that players were fully engaged. To execute a fast tennis serve, several factors are crucial: well‐coordinated joints' movement from proximal to distal segments (i.e., lower limbs, trunk and then upper limbs) for optimal velocity summation, achieving necessary ranges of motion through the kinetic chain, and maintaining consistency in key serve events (Colomar, Corbi, & Baiget, 2022; Colomar, Corbi, Brich, & Baiget, 2022). In our study, preserved maximum ball velocity and peak vertical force data suggest that compensatory adaptations in hip and trunk rotations, upper‐arm extension, and internal rotation help maintain racket and ball velocity following prolonged play. However, 3D marker‐based motion capture systems, while being the gold standard, are time‐consuming and costly and were not used here. Recent advancements in vision and machine learning now offer the potential to assess these adjustments using video‐based, multi‐camera, marker‐less systems (Colyer et al., 2018).

Regardless of measurement time point, all muscles exhibited higher numerical EMG values for the first compared to the second serve. Nonetheless, out of eight studied muscles, this difference was only significant for half of them (i.e., left *rectus femoris* and *gastrocnemius lateralis* muscles and right *vastus lateralis* and *gastrocnemius lateralis* muscles). Overall, these changes were more pronounced in the first serve (ranging from −10% to −40%) compared to the second serve (ranging from 0% to −25%). The magnitude of changes in the EMG signal aligns with those reported for first serves throughout a three‐set tennis match, where reductions of ∼50% (in the *rectus femoris* muscle at the end of set 2) or even slightly exceeding this value (in the *biceps femoris* muscle at the end of set 3) have been documented by Fenter et al. (2017). However, the limitation of collecting data for the back leg only and not controlling for the type of serve hinders the strength of comparisons.

### 4.3 Compensatory strategies

According to Rota et al. (2014), different compensation strategies can be devised to redistribute muscle activity to non‐fatigued muscles, alleviating the decline in performance. These authors argued that a strenuous tennis exercise preferentially induces adjustments in the muscle activity levels of several upper limb muscles rather than changes in the modular organization of muscle coordination. Aune et al. (2008) provided supporting evidence, demonstrating that expert table tennis players use adjustment strategies to compensate for fatigue during attacking forehands, whereas recreational players exhibit decreased accuracy. Similarly, higher‐skilled tennis players demonstrate better maintenance of elbow joint, wrist joint, and racket handle stability when fatigued, leading to enhanced serve efficacy compared to their less skilled counterparts (Wang et al., 2021). Collectively, these findings indicate that skilled players, as examined in this study, have developed the ability to adapt motor coordination strategies in response to a 3‐h tennis match.

Consistent trends in either higher or lower EMG values were not observed when comparing the left (front) and right (back) legs. Furthermore, there was no indication of preferential alterations in the quadriceps or ankle muscles due to prolonged play, whether during first or second serves. One explanation is the recruitment of well‐trained tennis players in our study. Higher skilled players exhibit greater resilience to fatigue‐induced alterations, thereby contributing to better preservation of stroke reliability and accuracy compared to their lower‐level counterparts (Lyons et al., 2013). The timing of muscle activation in lower limb muscles has been identified as a key distinction between elite and lower‐level players, demonstrating more precocious periods of high muscular activity during the acceleration phase of the serve in elite players when tested in unfatigued conditions (Girard et al., 2005). However, relying solely on EMG values is insufficient for interpreting the origin of fatigue (i.e., without percutaneous nerve stimulations; Girard et al., 2008, 2011) and inferring changes in segmental reorganization (i.e., in the absence of kinematic analysis; Colomar, Corbi, & Baiget, 2022; Colomar, Corbi, Brich, & Baiget, 2022). In a previous study, a 3‐h tennis match was shown to significantly decrease serve ball speed, ball impact height, and maximal knee and upper limb angular velocities as well as decrease or maintain upper limb joint kinetics in advanced male tennis players (Martin et al., 2016). However, without kinematic data in our study, additional research is necessary to confirm whether adjustments in lower limb muscular activity following a prolonged tennis match disrupt the kinematic chain or not.

### 4.4 Interindividual variability

Considerable interindividual variability was observed among all muscles, regardless of measurement timing or serve type. Generally, the EMG values for most individuals were within approximately twice the magnitude of the mean value for a given muscle. In addition to the inherent limitations in interpreting signal amplitudes using surface EMG techniques (Vigotsky et al., 2018), inconsistencies in the EMG signal may also be attributed to natural compensatory strategies, wherein various neuromuscular actions could be employed to maintain peak vertical force. Numerous studies have mentioned important intraindividual and interindividual variability in EMG patterns during serve actions (Chow et al., 2003; Miyashita et al., 1980), suggesting that the consistency of the EMG signals may also be considered, notably when assessing fatigue effects. Our study focused on interindividual variability rather than intraindividual variability, analyzing only five serves per participant to avoid excessive fatigue, which limited the number of trials per individual.

### 4.5 Limitations and additional considerations

This study is not without limitations. Firstly, we recorded only five successful first and second tennis serves, which might be considered relatively limited but is consistent with previous EMG studies on tennis serves (Fenter et al., 2017). This methodological decision aims at preventing excessive recovery to capture the effects of extended play. Secondly, first and second serve biomechanics were assessed from male competitive players on a hard surface. A recent scoping review revealed a scarcity of data regarding kick and slice serves, foot‐back and foot‐up serves, clay and grass surfaces, and left‐handed, female, and young (12–17 years) player analyses (Brito et al., 2023). Therefore, extending our findings to other demographics and playing conditions may have only partial applicability at best. Thirdly, our sample size (*n* = 10) was rather small. However, since the participants were all highly ranked tennis players, considered a homogeneous group in terms of serving skill and a within‐subject design was used, the small sample size should not significantly impact the major findings of this study.

Several other methodological considerations need to be taken into account while interpreting findings. While this study focused on the effects of a 3‐h tennis match, it is important to acknowledge that temporary fatigue from intense rallies, even in fresh conditions (e.g., during the first set of a contested game), could result in distinct neuromechanical adjustments during the serve. This possibility is indirectly supported by the observed high peak heart rate values (∼180 beat/min) during the games, which also confirms the participants' strong motivation to compete at a high level. In their study aiming to simulate important periods of the match involving crucial points, such as the end of a set or a tie‐break, Rota et al. (2014) reported a decrease in the EMG activation level of *pectoralis major* and *flexor carpi radialis* muscles that was accompanied by a 4.5% reduction of maximum ball velocity. We also acknowledge that several factors, including current score, crowd, expectations, and psychological stress, are likely to influence psychophysiological responses during actual tournaments scenarios. However, the impact of these stressors on neuromechanical adjustments of the serve remains unknown. Finally, in our study, participants served to the “deuce” box where the force plate was located. Since changing the service side can affect serve and ball kinematics (Fett et al., 2021), caution is required in generalizing results to serves made to the “advantage” box.

### 4.6 Practical implications

A tennis match involves hundreds of serves, making the maintenance of serve efficacy paramount, notably to win decisive points. In their retrospective analysis of fifty 5‐set matches of the 2014 Grand Slam tournaments, Martin et al. (2019) noted a significant increase (∼5 km/h) in first serve velocity in the fifth set for winners, compared to losers. To date, there are robust evidences advocating for tailored conditioning programs to enhance lower limb muscular strength through resistance training, leading to improved skilled performance. This enhancement is demonstrated, for example, by a more forceful leg drive, leading to higher ball‐racket contact height and subsequently faster serve velocities (Brito et al., 2023; Colomar, Corbi, & Baiget, 2022; Colomar, Corbi, Brich, & Baiget, 2022). Understanding reported changes in muscle activation on each leg can serve as a reference for expected alterations in muscle activation during first and second serves, especially after extended play. This knowledge can assist trainees and coaches in maintaining an effective serve technique following prolonged tennis playing by adopting neuromechanical adjustment strategies.

## 5 CONCLUSION

We compared surface EMG activity in eight lower limb muscles during first and second serves before and after prolonged match play tennis. Our main observation is the selective reduction in activity levels of both knee extensor and plantar flexor muscles after a 3‐h tennis match, despite unchanged maximum ball velocity and peak vertical forces. Specifically, this alteration affects the *vastus lateralis*, *gastrocnemius lateralis*, and *soleus* muscles in the left leg as well as the *vastus lateralis* muscle in the right leg. Additionally, the type of serve influenced reductions in EMG activity levels, with generally more pronounced changes observed for first serves compared to second serves. This information supports the concept of compensatory muscle activation strategies during the serve, highlighting the need for future research to elucidate segment coordination patterns following extended play.

## CONFLICT OF INTEREST STATEMENT

The authors declare that they have no conflicts of interest.
